# Advances in Nonnegative Matrix and Tensor Factorization

**DOI:** 10.1155/2008/852187

**Published:** 2008-07-06

**Authors:** A. Cichocki, M. Mørup, P. Smaragdis, W. Wang, R. Zdunek

**Affiliations:** ^1^Laboratory for Advanced Brain Signal Processing, RIKEN Brain Science Institute, Saitama 351-0198, Japan; ^2^Department of Informatics and Mathematical Modeling, Technical University of Denmark, Richard Petersens Plads, Building 321, 2800 Lyngby, Denmark; ^3^Advanced Technology Labs, Adobe Systems Inc., 275 Grove Street, Newton, MA 02466, USA; ^4^Centre for Vision, Speech, and Signal Processing, University of Surrey, Guildford GU2 7XH, UK; ^5^Institute of Telecommunications, Teleinformatics, and Acoustics, Wroclaw University of Technology, Wybrzeze Wyspianskiego 27, 50370 Wroclaw, Poland

Nonnegative matrix factorization (NMF) and its extension known as nonnegative tensor factorization (NTF) are emerging techniques that have been proposed recently. The goal of NMF/NTF is to decompose a nonnegative data matrix into a product of lower-rank nonnegative matrices or tensors (i.e., multiway arrays). An NMF approach similar to independent component analysis (ICA) or sparse component analysis (SCA) is very useful and promising for decomposing high-dimensional datasets into a lower-dimensional space. A great deal of interest has been given very recently to NMF models and techniques due to their capability of providing new insights and relevant information on the complex latent relationships in experimental datasets, and due to providing meaningful components with physical or physiological interpretations. For example, in bioinformatics, NMF and its extensions have been successfully applied to gene expression, sequence analysis, functional characterization of genes, clustering, and text mining. The main difference between NMF and other classical factorizations such as PCA, SCA, or ICA methods relies on the nonnegativity, and usually also additional constraints such as sparseness, smoothness, and/or orthogonality imposed on the models. These constraints tend to lead to a parts-based representation of the data, because they allow only additive, not subtractive, combinations of data items. In this way, the nonnegative components or factors produced by this approach can be interpreted as parts of the data. In other words, NMF yields nonnegative factors, which can be advantageous from the point of view of interpretability of the estimated components. Furthermore, in many real applications, data have a multiway (multiway array or tensor) structure. Exemplary data are video stream (rows, columns, RGB color coordinates, time), EEG in neuroscience (channels, frequency, time, samples, conditions, subjects), bibliographic text data (keywords, papers, authors, journals), and so on. Conventional methods preprocess multiway data, arranging them into a matrix. Recently, there has been a great deal of research on multiway analysis which conserves the original multiway structure of the data. The techniques have been shown to be very useful in a number of applications, such as signal separation, feature extraction, audio coding, speech classification, image compression, spectral clustering, neuroscience, and biomedical signal analysis.

This special issue focuses on the most recent advances in NMF/NTF methods, with emphasis on the efforts made particularly by the researchers from the signal processing and neuroscience area. It reports novel theoretical results, efficient algorithms, and their applications. It also provides insight into current challenging areas, and identifies future research directions.

This issue includes several important contributions which cover a wide range of approaches and techniques for NMF/NTF and their applications. These contributions are summarized as follows.

The first paper, entitled “Probabilistic latent variable models as nonnegative factorizations” by M. Shashanka et al., presents a family of probabilistic latent variable models that can be used for analysis of nonnegative data. The paper shows that there are strong ties between NMF and this family, and provides some straightforward extensions which can help in dealing with shift invariances, higher-order decompositions, and sparsity constraints. Furthermore, it argues through these extensions that the use of this approach allows for rapid development of complex statistical models for analyzing nonnegative data.

The second paper, entitled “Fast nonnegative matrix factorization algorithms using projected gradient approaches for large-scale problems” by R. Zdunek and A. Cichocki, investigates the applicability of projected gradient (PG) methods to NMF, based on the observation that the PG methods have high efficiency in solving large-scale convex minimization problems subject to linear constraints, since the minimization problems underlying NMF of large matrices well match this class of minimization problems. In particular, the paper has investigated several modified and adopted methods, including projected Landweber method, Barzilai-Borwein gradient projection, projected sequential subspace optimization, interior-point Newton algorithm, and sequential coordinatewise minimization algorithm, and compared their performance in terms of signal-to-interference ratio and elapsed time, using a simple benchmark of mixed partially dependent nonnegative signals.

The third paper, entitled “Theorems on positive data: on the uniqueness of NMF” by H. Laurberg et al., investigates the conditions for which NMF is unique, and introduces several theorems which can determine whether the decomposition is in fact unique or not. Several examples are provided to show the use of the theorems and their limitations. The paper also shows that corruption of a unique NMF matrix by additive noise leads to a noisy estimation of the noise-free unique solution. Moreover, it uses a stochastic view of NMF to analyze which characterization of the underlying model will result in an NMF with small estimation errors.

The fourth paper, entitled “Nonnegative matrix factorization with Gaussian process priors” by M. N. Schmidt and H. Laurberg, presents a general method for including prior knowledge in NMF, based on Gaussian process priors. It assumes that the nonnegative factors in the NMF are linked by a strictly increasing function to an underlying Gaussian process specified by its covariance function. The NMF decompositions are found to be in agreement with the prior knowledge of the distribution of the factors, such as sparseness, smoothness, and symmetries.

The fifth paper, entitled “Extended nonnegative tensor factorisation models for musical sound source separation” by D. FitzGerald et al., presents a new additive synthesis-based NTF approach which allows the use of linear-frequency spectrograms as well as imposing strict harmonic constraints, resulting in an improved model as compared with some existing shift-invariant tensor factorization algorithms in which the use of log-frequency spectrograms to allow shift invariance in frequency causes problems when attempting to resynthesize the separated sources. The paper further studies the addition of a source filter model to the factorization framework, and presents an extended model which is capable of separating mixtures of pitched and percussive instruments simultaneously.

The sixth paper, entitled “Gene tree labeling using nonnegative matrix factorization on biomedical literature” by K. E. Heinrich et al., addresses a challenging problem for biological applications, that is, identifying functional groups of genes. It examines the NMF technique for labeling hierarchical trees. It proposes a generic labeling algorithm as well as an evaluation technique, and discusses the effects of different NMF parameters with regard to convergence and labeling accuracy. The primary goals of this paper are to provide a qualitative assessment of the NMF and its various parameters and initialization, to provide an automated way to classify biomedical data, and to provide a method for evaluating labeled data assuming a static input tree. This paper also proposes a method for generating gold standard trees.

The seventh paper, entitled “Single-trial decoding of bistable perception based on sparse nonnegative tensor decomposition” by Z. Wang et al., presents a sparse NTF-based method to extract features from the local field potential (LFP), collected from the middle temporal visual cortex in a macaque monkey, for decoding its bistable structure-from-motion perception. The advantages of the sparse NTF-based feature-extraction approach lie in its capability to yield components common across the space, time, and frequency domains, yet discriminative across different conditions without prior knowledge of the discriminating frequency bands and temporal windows for a specific subject. The results suggest that imposing the sparseness constraints on the NTF improves extraction of the gamma band feature which carries the most discriminative information for bistable perception.

The eighth paper, entitled “Pattern expression nonnegative matrix factorization: algorithm and applications to blind source separation” by J. Zhang et al., presents a pattern expression NMF (PE-NMF) approach from the view point of using basis vectors most effectively to express patterns. Two regularization or penalty terms are introduced to be added to the original loss function of a standard NMF for effective expression of patterns with basis vectors in the PE-NMF. A learning algorithm is presented, and the convergence of the algorithm is proved theoretically. Three illustrative examples for blind source separation including heterogeneity correction for gene microarray data indicate that the sources can be successfully recovered with the proposed PE-NMF when the two parameters can be suitably chosen from prior knowledge of the problem.

The last paper, entitled “Robust object recognition under partial occlusions using NMF” by D. Soukup and I. Bajla, studies NMF methods for recognition tasks with occluded objects. The paper analyzes the influence of sparseness on recognition rates for various dimensions of subspaces generated for two image databases, ORL face database, and USPS handwritten digit database. It also studies the behavior of four types of distances between a projected unknown image object and feature vectors in NMF subspaces generated for training data. In the recognition phase, partial occlusions in the test images have been modeled by putting two randomly large, randomly positioned black rectangles into each test image.

